# Combined effect of pantoprazole and mesenchymal stem cells on experimentally induced gastric ulcer: implication of oxidative stress, inflammation and apoptosis pathways

**DOI:** 10.1007/s10787-024-01469-0

**Published:** 2024-04-23

**Authors:** Alaa H. Sayed, Nadia S. Mahmoud, Ola A. M. Mohawed, Hanaa H. Ahmed

**Affiliations:** 1 Hormones Department, Medical Research and Clinical Studies Institute, National Research Centre, Dokki 12622, Giza, Egypt; 2 Stem Cell Lab, Center of Excellence for Advanced Sciences, National Research Centre, Dokki, Giza, Egypt

**Keywords:** Gastric ulcer, Inflammation, Mesenchymal stem cells, Oxidative stress, Pantoprazole, Rats

## Abstract

Gastric ulcer (GU) is one of the most common diseases of the upper gastrointestinal tract that affects millions of people worldwide. This study aimed to investigate the possible alleviating effect of a combined treatment of pantoprazole (PANTO) and adipose tissue-derived mesenchymal stem cells (ADSCs) in comparison with each treatment alone on the healing process of the experimentally induced GU in rats, and to uncover the involved pathways. Rats were divided into five groups: (1) Control, (2) GU, (3) PANTO, (4) ADSCs and (5) ADSCs + PANTO. Markers of oxidative stress, inflammation and apoptosis were assessed. The current data indicated that PANTO-, ADSCs- and ADSCs + PANTO-treated groups showed significant drop (*p* < *0.05*) in serum advanced oxidation protein products (AOPPs) and advanced glycation end products (AGEPs) along with significant elevation (*p* < *0.05*) in serum TAC *versus* the untreated GU group. Moreover, the treated groups (PANTO, ADSCs and ADSCs + PANTO) displayed significant down-regulation (*p* < *0.05*) in gastric nuclear factor kappa-light-chain-enhancer of activated B cells (NF-κB), tumor necrosis factor alpha (TNF-α), cyclooxygenase-2 (COX-2), intercellular adhesion molecule-1 (ICAM-1), matrix metallopeptidase 9 (MMP*-*9) and caspase-3 along with significant up-regulation (*p* < *0.05*) in vascular endothelial growth factor (VEGF) and peroxisome proliferator-activated receptor gamma (PPARγ) genes expression compared to the untreated GU group. Immunohistochemical examination of gastric tissue for transforming growth factor β1 (TGF-β1), epidermal growth factor (EGF) and proliferating cell nuclear antigen (PCNA) showed moderate to mild and weak immune reactions, respectively in the PANTO-, ADSCs- and ADSCs + PANTO-treated rat. Histopathological investigation of gastric tissue revealed moderate to slight histopathological alterations and almost normal histological features of the epithelial cells, gastric mucosal layer, muscularis mucosa and submucosa in PANTO-, ADSCs- and ADSCs + PANTO-treated rats, respectively. Conclusively, the co-treatment with ADSCs and PANTO evidenced sententious physiological protection against GU by suppressing oxidative stress, inhibiting inflammation and reducing apoptosis with consequent acceleration of gastric tissue healing process.

## Introduction

Gastric ulcer (GU) is a common digestive tract disease with a long course, difficult treatment and a high recurrence rate that causes a serious burden on the public health system and living quality (Ma et al. 2022). It is an erosion in the gastrointestinal wall that may extend through the mucosal muscles into the deep layers of the gastric wall. It may also affect a major blood vessel resulting in life-threatening hemorrhage (Yeomans and Naesdal 2008). Anorexia, nausea, vomiting, bloating, gastrointestinal pain, and acid reflux are some of the common clinical signs of GU (Xie et al. 2021). According to Woolf and Rose (2021), gastric ulcer afflicts 8% to 10% of the global population. Nearly 40% of people in developed countries and 80% of people in developing countries have problems with GU (Alam et al. 2019).

The etiology of GU is multi-factorial as it can be triggered by the imbalance between the defending factors including nitric oxide, mucin, prostaglandins, the mucosal blood flow, and the capacity for epithelial regeneration on one side, and the destructive factors including smoking, NSAIDs, alcohol consumption, pepsin secretion, bile salts, gastric acid, and H. pylori contagion on the other side (Abd-Elhamid 2021). It has been demonstrated that 43% of people frequently take NSAIDs, and almost 30% of those subjects have gastric disorders (Bindu et al. 2020). IND is one of the classic NSAIDs that is used frequently in the treatment of inflammatory disorders. IND has been found to increase gastric acid secretion, decrease the level of PGE2 (AlKreathy et al. 2020), trigger oxidative stress (Hafez et al. 2019), inflammation and apoptosis in gastric tissue, resulting in GU (El Badawy et al. 2021). Therefore, numerous researches have established IND to induce GU in rats as a standard model of GU in humans (Gomaa et al. 2018).

The pharmacological treatment of GU includes antacids, muscarinic receptor antagonists, PPIs, histamine (H2) receptor antagonists, and H. pylori eradication therapies. These medications have certainly reduced the frequency of GU, however, they have a high risk of recurrence, drug interactions, and side effects (Johnley et al. 2020). PPIs have a powerful healing effect on GU as they block the secretion of gastric acid by hindering the proton pump H + K + ATPase in the gastric parietal cells (Alazzouni et al. 2020). Nevertheless, they can result in various side effects such as bacterial overgrowth within the gastric mucosa and/or enterochromaffin-like cell carcinoid as a result of hypergastrinemia (da Luz et al. 2021). Accordingly, research is directed more toward identifying potentially active candidates that may provide a safe and effective therapy for GU.

MSCs are multipotent cells with self-regeneration and differentiation capacity into multiple cell types making them superior in tissue damage repair and regeneration (Fu et al. 2019). The fact that ADSCs can be isolated with minimal morbidity and without any ethical concerns makes them attractive candidates for many clinical applications, including tissue healing and regeneration (Shingyochi et al. 2015). Recently, ADSCs have been found to induce vascularization, enrich in vivo stem cell microenvironment, diminish oxidative stress, and mediate immune reactions (Luck et al. 2019). The study by Xia et al. (2019) suggested that ADSCs infusion represents a hopeful strategy to stimulate the healing of NSAID-induced peptic ulceration via hampering inflammation and stimulating cellular proliferation and angiogenesis. Hence, the objectives of this research were to: (1) explore the therapeutic outcome of a combined treatment of pantoprazole and adipose tissue-derived mesenchymal stem cells in combating indomethacin-induced gastric ulcer in rats. (2) appraise the possible underlying mechanisms implicated in such therapeutic approach. This study will pave the way to disclose a new therapeutic modality for the complete curing of gastric ulcer with minimal side effects in a short duration.

## Material and methods

### Drugs and chemicals

Indomethacin (IND) (Liometacen 25 mg/ml) was purchased from El-Nile Co. for Pharmaceutical and Chemical Industries, Cairo, Egypt, and Pantoprazole (PANTO) (Pantoloc 40 mg) was procured from Medical Union Pharmaceuticals (MUP), Cairo, Egypt. All the other unspecified chemicals were of analytical grade.

### isolation and proliferation of adipose tissue-derived mesenchymal stem cells (ADSCs)

Adipose tissue was isolated from the inguinal and abdominal fat pads of female *Wistar* rats (120–150 g), procured from the Animal Care Facility unit of the National Research Centre, Egypt, after subjecting to general anesthesia according to the protocol described by Tomiyama et al. (2008). The fat tissue was then sliced and washed with phosphate-buffered saline (PBS; Biowest, France). After that, collagenase type II (0.075%, Serva Electrophoresis GmbH, Germany) was added to the shredded fat tissue with continuous shaking at 37 °C for 1 h. The resultant digested fat tissue was then filtrated and centrifuged at 400 *g* for 10 min at 25 °C. Erythrocyte-lysis buffer was used to eradicate any erythrocytes. The obtained cell pellet was suspended in Low glucose Dulbecco's modified Eagle’s medium (Lonza, Belgium) provided with 10% fetal bovine serum (FBS; Biowest, France) and 1% penicillin–streptomycin (Biowest, France), followed by incubation at 37 °C in a 5% CO_2_ incubator (Sartorius, Germany). The next day, the non-adherent cells were removed by replacing the old culture medium with a new one. Once ADSCs reached 90% confluence, they were propagated using 1X trypsin/EDTA (Biowest, France) for 5 min at 37 °C. Cell subculture was maintained till attaining third-passage cultures.

### Validation of ADSCs characteristics

ADSCs were identified by their spindle shape upon investigation under the inverted microscope and through screening their phenotypes by analyzing the MSCs-surface markers (CD90 and CD105) and a hematopoietic stem cells marker (CD34) (Woodbury et al. 2000). In brief, ADSCs were suspended in PBS supplemented with 0.5% bovine serum albumin (BSA)/2 mmol EDTA. After that, they were analyzed for surface markers expression using anti-rat fluorescein isothiocyanate (FITC)-labeled CD90 antibody (R&D Systems, UK), anti-rat FITC-conjugated CD105 antibody (MiltenyiBiotec, Germany), anti-rat FITC-labeled CD34 antibody (Beckman Coulter Co., USA). Then, the cells were washed twice with PBS supplied with 2% BSA, re-suspended in PBS, and analyzed using flow cytometry (Beckman Coulter Elite XL, USA instrument). Isotype control matched rat immunoglobulin was used for auto-fluorescence. Furthermore, ADSCs tri-lineage differentiation ability was evaluated through inducing their differentiation into adipogenic, chondrogenic and osteogenic lineages using StemPro® differentiation medium according to the associated protocols.

#### ADSCs differentiation into adipocytes

In brief, ADSCs of the 3rd passage (5 × 10^5^/well) were cultivated in a 6-well culture plate and incubated at 37 °C. Once the cells reached 90% confluence, the growth media were replaced by StemPro® adipogenic media (Cat. No. A1007001, Gibco, Thermo Fisher Scientific Inc., USA). Cells were incubated at 37 °C in a 5% CO_2_ incubator and the adipogenic media were replaced every 3 days for 15 days. Control wells represented cells cultured in a growth medium consisting of LG-DMEM supplied with 10% FBS and 1% penicillin/streptomycin. After the adipogenic differentiation, 10% formalin solution was added for cell fixation for 30 min, followed by twice washing with PBS and staining with Oil Red O (Sigma-Aldrich, St. Louis, MO, USA), and then incubation for 15 min at room temperature away from light. Lastly, cells were investigated under the inverted microscope (Olympus, Japan) to detect the intracellular lipid droplets.

#### ADSCs differentiation into chondrocytes

Briefly, 5 × 10^5^ ADSCs/well were plated into a 6-well culture plate till obtaining 90% confluence. Then, the growth media were replaced with StemPro® chondrogenesis differentiation medium (Cat. No. A1007101, Gibco, Thermo Fisher Scientific Inc., USA). Cells were then incubated at 37 °C with 5% humidified CO_2_ and the media were changed every 3 days for 15 days. Control wells denoted cells cultured in LG-DMEM provided with 10% FBS and 1% penicillin/streptomycin. After chondrogenic differentiation, cell fixation was performed with 10% formalin solution for 30 min. Following fixation, cells were rinsed twice with PBS and stained with Alcian Blue (1%, Sigma-Aldrich, St. Louis, MO, USA) and incubated for 15 min at room temperature in the dark. After that, cells were examined under the inverted microscope to determine the formation of the blue-stained glycosaminoglycan.

#### ADSCs differentiation into osteocytes

ADSCs were motivated to differentiate into osteocytes using StemPro® osteogenic medium (Cat. No. A1007201, Gibco, Thermo Fisher Scientific Inc., USA). Cells suspended in LG-DMEM supplied with 10% FBS and 1% penicillin/streptomycin were assigned as control. After 21 days of osteogenic differentiation, both undifferentiated and differentiated cells were fixed with 10% formalin solution for 30 min. After that, the wells were washed with PBS and then stained with Alizarin Red S (pH 4.2) (2%, Sigma-Aldrich, St. Louis, MO, USA) for 15 min. Finally, the formation of calcium nodules was investigated under the inverted microscope.

### Animal experiment

#### Animals

Thirty-five adult female albino *Wistar* rats, aged 3 months and weighing 120–140 g were obtained from the Animal Care Unit of the National Research Centre (NRC), Egypt, and housed in the Sectorial Animal Facility of Hormones Department, Medical Research and Clinical Studies Institute, NRC under controlled room temperature (27 ± 3 °C) and a 12 h/12 h light/dark cycle. Animals were maintained in plastic cages with wood shavings used as beddings, and provided with standard food and water ad libitum*.* The animals were kept under observation for about one week before the onset of the experiment for adaptation. The experimental protocol was performed as per Institutional guidelines as the ethics committee approval was obtained from the Ethical Committee for Medical Research, NRC, Egypt for all the procedures with animals in this study (Decision number 3436072022).

#### Experimental protocol

After the habituation period, the rats were fasted for 12 h and then allocated to five groups (7 rats/group) as follows—Group 1: the negative control group; this group was intraperitoneally injected with vehicle (phosphate buffer saline; PBS) once at the beginning of the experiment. Group 2: the GU group; this group was intraperitoneally injected with IND (25 mg/kg) (AlKreathy et al. 2020) once at the beginning of the experiment for induction of GU. Then, 4 h later, the blood samples were collected from the tail vein and the sera were separated for biochemical determinations. After that, the rats were sacrificed, and each stomach was dissected for histopathological examination, immunohistochemical investigation and molecular genetics analyses. Group 3: the PANTO group; this group was intraperitoneally injected with IND (25 mg/kg) once at the beginning of the experiment for induction of GU. Then, 4 h later, the rats were orally gavaged with PANTO (20 mg/kg) daily for 2 weeks (Talaat et al. 2014). Group 4: the ADSCs group; this group was intraperitoneally injected with IND (25 mg/kg) once at the beginning of the experiment. Then, 4 h later, the rats were intravenously infused with a single dose of ADSCs (1 × 10^6^ cells)/rat (El Kasaby et al. 2017) and left for 2 weeks. Group 5: the ADSCs + PANTO group; this group was intraperitoneally injected with IND (25 mg/kg) once at the beginning of the experiment. Then, 4 h later, the rats were infused intravenously with a single dose of ADSCs (1 × 10^6^ cells)/rat and received an oral daily dose of PANTO (20 mg/kg) for 2 weeks.

### Blood and tissue samples collection

At the end of the experiment (2 weeks), all rats (except for Group 2 which was sacrificed after 4 h from induction of GU) were allowed to fast overnight. Following full anesthesia via i.p. injection of ketamine 90 mg/kg and xylazine 5 mg/kg (Othman et al. 2021), blood samples were collected from the tail vein, and the sera were separated by allowing the blood samples to clot for 30 min at temperature of 25 °C, and then centrifuged at 1800 *g* for 10 min at 4 °C (). After centrifugation, the serum samples were separated into numerous aliquots and cryopreserved until further usage in serological evaluation.

After blood collection, the rats were rapidly sacrificed by cervical dislocation, and the stomach of each rat was dissected, cleaned, washed with ice-cold saline (0.9% NaCl), and, then, a portion of the gastric tissue was immediately preserved in liquid nitrogen and kept at −80 °C for molecular genetics analyses. The rest of the gastric tissue was fixed in 10% formalin saline for immunohistochemical and histopathological examinations.

### Serological evaluation

Serum TAC was spectrophotometrically measured according to the manufacturer’s guidelines provided with the kit (Cat. NO. TA2513) purchased from Biodiagnostic Co. (Giza, Egypt). Serum AGEPs were quantified according to the manufacturer’s manual of the enzyme-linked immunosorbent assay (ELISA) kit (Cat No. MBS261131) procured from MyBioSource Co. (San Diego, USA). Serum AOPPs was estimated according to the manufacturer’s recommendation of the ELISA kit (Cat No. CSB-EQ027429RA) obtained from Cusabio Co. (Houston, USA). Briefly, serum samples (100 μL) were added to a 96-well plate coated with an antibody specific to the marker being investigated at 37 °C for 90 min, and incubated with the specific diluted biotinylated rat antibody (100 μL) and incubated at 37 °C for 60 min. Avidin-HRP conjugate liquid (100 μL) was added to the 96-well plate followed by incubation at 37 °C for 30 min with several washes in between. The color reagent liquid (100 μL) was added in the dark and incubated at 37 °C for 15–30 min. Lastly, the color reagent C was added to individual wells and the absorbance was determined at 450 nm.

### Molecular genetic analyses

#### Quantitative analysis of gastric-ulcer-related gene expression

RNA was extracted from the stomachs of the rats using an RNeasy mini kit (Cat. #74,104, Qiagen, Germany). The obtained RNA was reverse transcribed into cDNA using a RevertAid cDNA synthesis kit (Cat# K1621, Thermo Fisher Scientific, Lithuania) according to the associated protocol. Gene expression levels of NF-κB, TNF-α, COX-2, MMP*-*9, ICAM-1, PPARγ, VEGF as well as Caspase-3 were analyzed using DNA-Technology Real-Time PCR device (DTlite 4, Russia). The PCR mixture (25 μl) included 12.5 μl QuantiTect SYBR Green master mix (Qiagen, Germany), 1 μl of each forward, and reverse primers of the studied genes (Invitrogen, USA), 100 ng of cDNA, and nuclease-free water. The relative mRNA expression levels *versus* the value of corresponding control was assessed using the 2^−ΔΔCt^ comparative method after normalization against GAPDH (Livak and Schmittgen 2001). The amplification program included one step of initial denaturation at 94 °C for 15 min, followed by 40 cycles (94 °C for 15 s, 60 °C for 30 s, and 72 °C for 30 s). The sequences of the primer pairs of studied genes are delineated in Table 1. Data were expressed as the fold change in the gene expression levels of the untreated gastric ulcer group relative to those of the negative control group. Data of all treated groups were represented as the fold change in gene expression relative to the untreated gastric ulcer group.

**Table 1:** List of primer sequences of genes used in qPCR

Genes	Primer sequences (5′–3′)	References
NF-κB	F: GCACGGATGACAGAGGCGTGTATAAGG	He et al. (2019)
	R: GGCGGATGATCTCCTTCTCTCTGTCTG	
TNF-α	F: CAGACCCTCACACTCAGATCATCTT	Hussien et al. (2018)
	R: CAGAGCAATGACTCCAAAGTAGACCT	
ICAM-1	F: CTCTGCTCCTGGTCCTGGT	Meng et al. (2016)
	R: CGTGAATGTGATCTCCTTGG	
COX-2	F: CTCCTTGAACACGGACTTGC	McCormick et al. (2010)
	R: TCAGGGAGAAGCGTTTGC	
MMP-9	F: GGATGTTTTTGATGCCATTGCTG	Luo et al. (2021)
	R: CCACGTGCGGGCAATAAGAAAG	
Caspase-3	F: TGGTACCGATGTCGATGCAGC	Saini et al. (2013)
	R: GGTCCACAGGTCCGTTCGTT	
VEGF	F: CACTGGACCCTGGCTTTACT	Shinagawa et al. (2019)
	R: GACGTCCATGAACTTCACCA	
PPARγ	F: TCTGGGAGATCCTCCTGTT	Lestari et al. (2015)
	R:CAATCGGATGGTTCTTCGGA	
GAPDH	F: CACCCTGTTGCTGTAGCCATATTC	Wu et al. (2007)
	R: GACATCAAGAAGGTGGTGAAGCAG	

### Immunohistochemical technique

After fixation of gastric tissues in 10% formalin saline overnight, the tissues were embedded in paraffin bee wax at 56 °C for 6 h. Paraffin wax tissues were cut by rotary microtome at 5 µm thickness. Then the sections were deparaffinized and were washed with tap water and dehydrated in serial dilutions of ethyl alcohol. Then the specimens were cleared in xylene and placed in 0.3% hydrogen peroxide/methanol for 20 min to block endogenous hydrogen peroxidase activity followed by washing with PBS. After that, 10% normal goat serum was added followed by incubation overnight at 4 °C in a humid chamber with mouse monoclonal anti- PCNA (1:75) (Santa Cruz Biotechnology), rabbit polyclonal anti-TGF-β1 (1:500) (Abcam, USA), and anti-EGF (1:100) (Abcam, USA). After that, they were incubated with the biotinylated goat anti-rabbit secondary antibody (1:200, Vector Labs, Peterborough, UK) for 30 min at room temperature followed by the addition of 3,3′-diaminobenzidine (DAB) chromogen. Eventually, sections were counterstained with Mayer’s hematoxylin and examined by a light microscope. For negative control; sections were routinely processed but the primary antibodies were replaced by PBS.

### Histopathological procedure

The injury of the stomach was evaluated by the histological examination of gastric tissue. The paraffin wax tissue blocks prepared for histopathological investigation were sectioned by using microtome at 5 µm thickness, and the sections were placed on glass slides, deparaffinized, and stained with hematoxylin–eosin (HE) staining for routine histopathological investigation under light microscope (Bancroft and Gamble 2008).

## Statistical analysis

Data were statistically analyzed using the statistical software, SPSS (version 20). The results were presented as mean ± SE. Significance was determined using one-way ANOVA test, *p* < 0.05 was considered significant.

## Results

### ADSCs identification

**Fig. 1:**
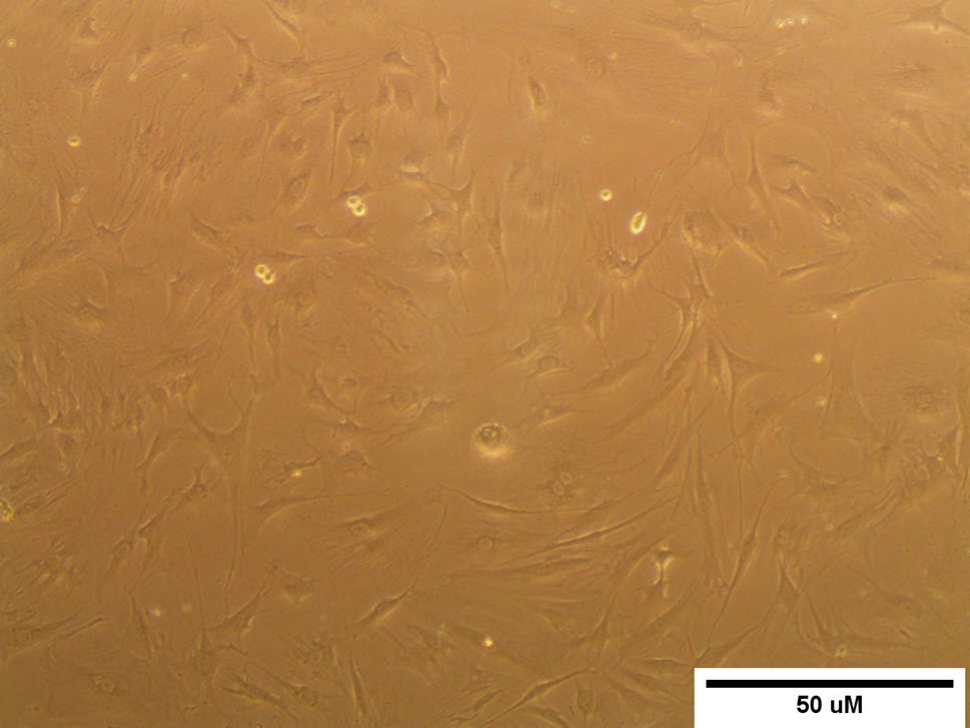
Microscopic features of 3rd passage ADSCs cultures showing adherent spindle shape (scale bar: 50 µm)

**Fig. 2:**
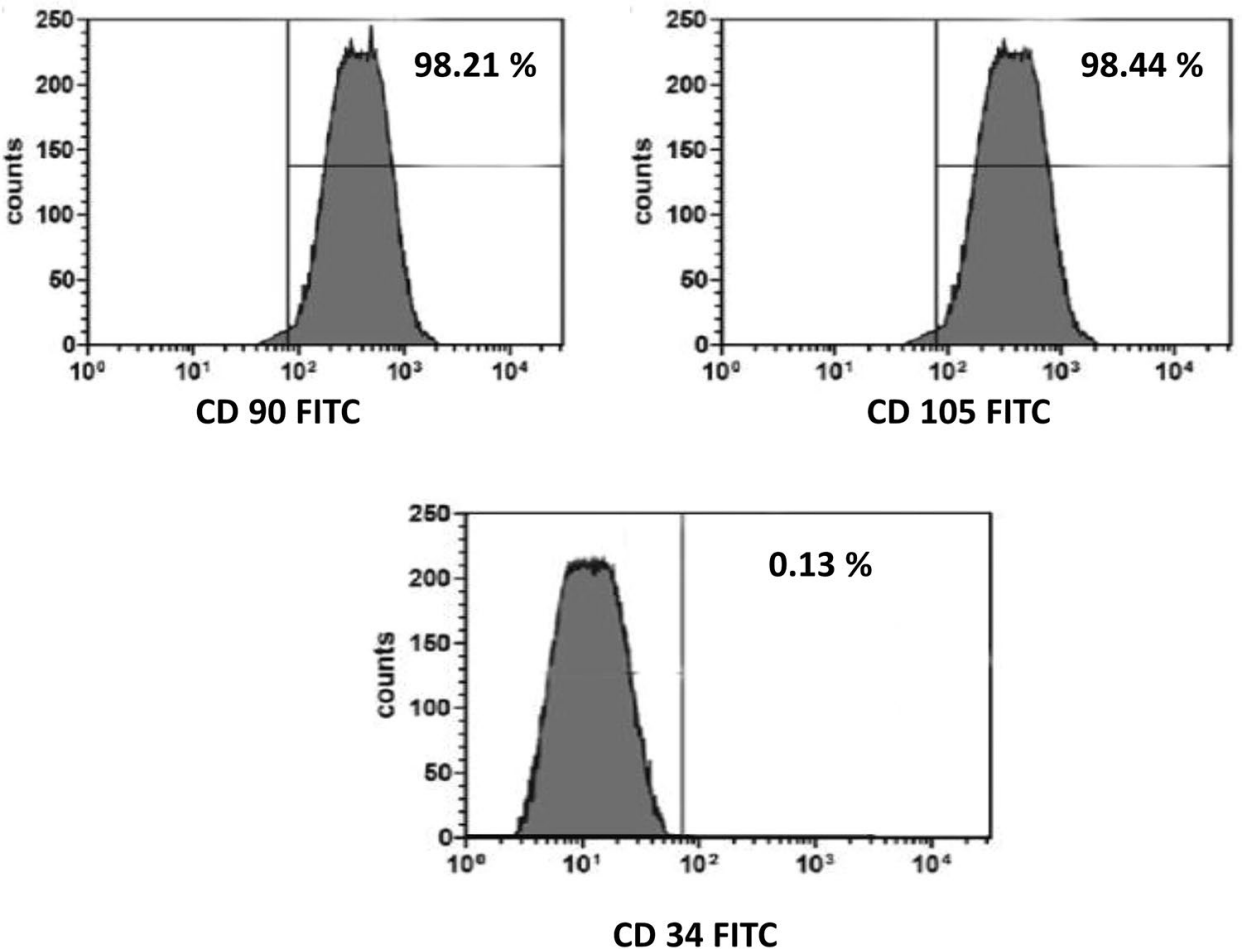
Flow cytometry characterization of ADSCs showing positive expression of MSCs-specific surface markers (CD90, CD105) and negative expression of hematopoietic-specific marker (CD34)

**Fig. 3:**
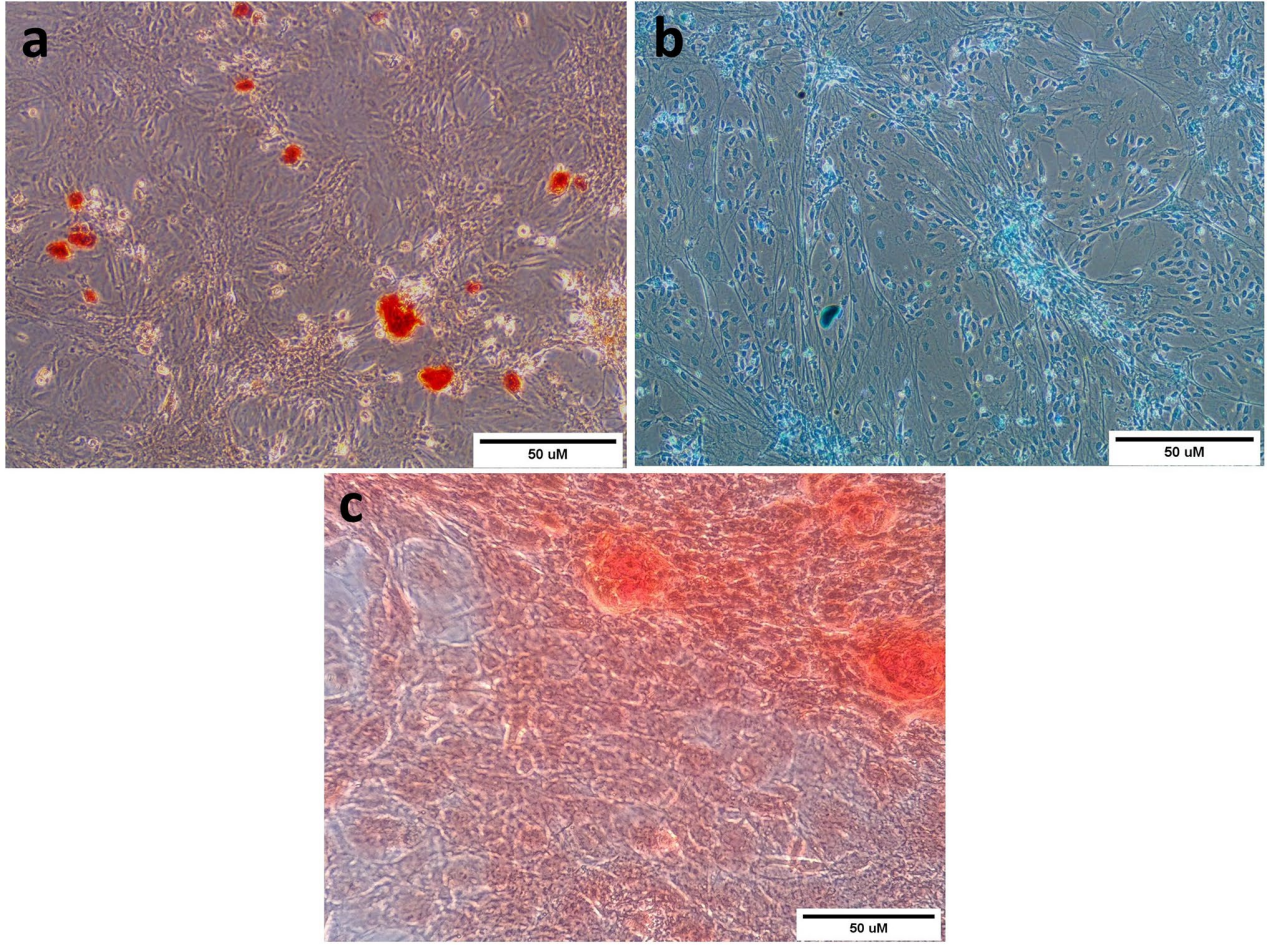
Tri-lineage differentiation potential of ADSCs into: (**a**) Adipocytes, as confirmed by Oil Red O staining of intracellular lipid droplets, (**b**) Chondrocytes, as confirmed by Alcian Blue staining of proteoglycan, and (**c**) Osteocytes, as confirmed by Alizarin Red staining of calcium deposits, (Scale bar: 50 µm)

Inverted microscope examination demonstrated the spindle morphology of ADSCs cultures, as shown in Fig. 1. Moreover, flow cytometry screening indicated that ADSCs displayed positive immunostaining for CD90 (98.21%), CD105 (98.44%), and negative staining for CD34 (0.13%) (Fig. 2). ADSCs were successfully differentiated into adipocytes following Oil Red O staining (Fig. 3a), whereas chondrogenic differentiation of ADSCs was documented by Alcian Blue staining (Fig. 3b). Furthermore, ADSCS differentiation into osteoblasts was affirmed by Alizarin Red S staining (Fig. 3c).

### Serological outcomes

#### The effect of ADSCs and PANTO on serum AOPPs level

The results tabulated in Table 2 reveal the influence of administration with PANTO and ADSCs, either alone or in combination, on serum AOPPs, TAC and AGEPs in GU-bearing rats. The induction of GU by IND resulted in a significant rise (*p* < *0.05*) in the serum AOPPs level relative to the negative controls. On the other side, the treated groups with PANTO, ADSCs or ADSCs + PANTO showed a significant drop (*p* < *0.05*) in the serum AOPPs level *versus* the GU group. Interestingly, the ADSCs-infused group showed a significant decrease (*p* < *0.05*) in the serum AOPPs level relative to the PANTO-administered group. Moreover, the serum AOPPs level revealed a significant decrease (*p* < *0.05*) in the ADSCs + PANTO-treated group compared with both the PANTO- and ADSCs-treated groups.

**Table 2:** Effect of ADSCs and PANTO either alone or in combination on the oxidant/antioxidant status of rats bearing GU induced by IND

Parameters groups	AOPPs (nmol/ml)	AGEPs (ng/ml)	TAC (mMol/L)
Negative control (NC)	216.1 ± 16.6	72.2 ± 2.4	49.9 ± 1.4
Gastric ulcer (GU)	1302.5 ± 30.2^a^	728.6 ± 60.5^a^	14.8 ± 1.2^a^
PANTO	993.4 ± 20.9^b^	455.8 ± 9.2^b^	25.4 ± 1.8^b^
ADSCs	756.2 ± 27.9^bc^	356.8 ± 21.7^bc^	29.7 ± 2.4^b^
ADSCs + PANTO	545.5 ± 19.1^bcd^	268.1 ± 21.3^bcd^	39.2 ± 0.3^bcd^

Data were represented as Mean ± S.E of 7 rats/group

^a^Significant difference at *P* < 0.05 relative to the negative control (NC) group

^b^Significant difference at *P* < 0.05 relative to the gastric ulcer (GU) group

^c^Significant difference at *P* < 0.05 relative to the PANTO-treated group

^d^Significant difference at *P* < 0.05 relative to the ADSCs-treated group

#### The effect of ADSCs and PANTO on serum AGEPs level

As demonstrated in Table 2, IND produced a significant increase in serum AGEPs level in the GU group *versus* the negative control group. On the contrary, the treated groups with PANTO, ADSCs or ADSCs + PANTO disclosed a significant decrease (*p* < *0.05*) in serum AGEPs level when compared with the GU group. Additionally, the ADSCs group exhibited a significant decrease (*p* < *0.05*) in serum AGEPs level contrary to the PANTO-treated group. In comparison with the ADSCs + PANTO-treated group, PANTO- or ADSCs-treated groups showed a significant increase (*p* < *0.05*) in serum AGEPs level.

#### The effect of ADSCs and PANTO on serum TAC:

As depicted in Table 2, in the GU group, IND significantly (*p* < 0.05) reduced the serum TAC by contrast with the negative control group. However, the treated groups with PANTO, ADSCs or ADSCs + PANTO brought about a significant increase (*p* < *0.05*) in serum TAC as compared to the GU group. Besides, the ADSCs + PANTO-treated group showed a significant increase (*p* < *0.05*) in serum TAC when compared with the PANTO and ADSCs-treated groups.

### Molecular genetics outputs

#### Gene expression of the inflammatory and anti-inflammatory markers

**Fig. 4:**
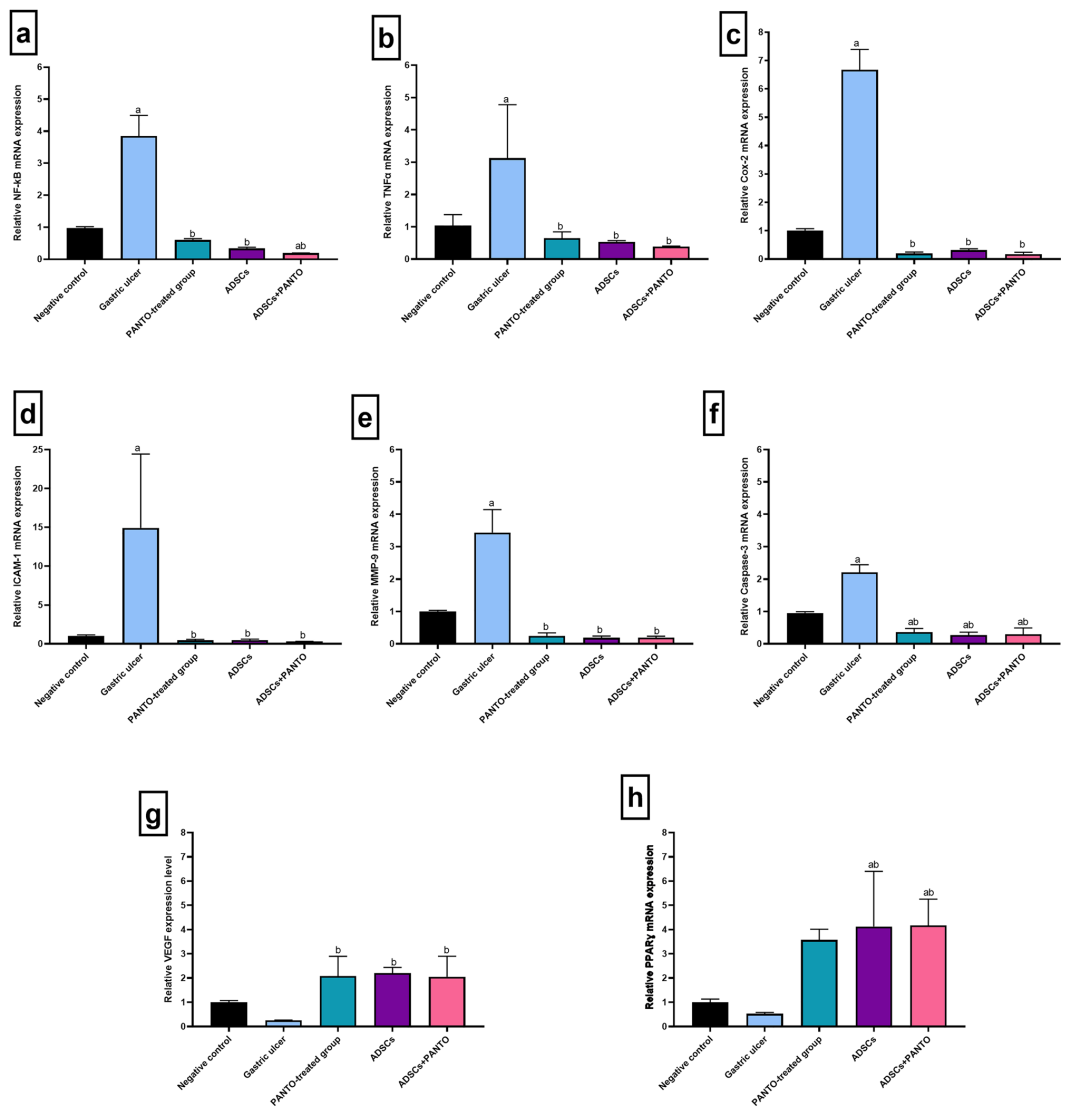
Effect of PANTO and ADSCs, either alone or in combination, on the gene expression levels of gastric ulcer-related genes by qRT-PCR. Relative expression levels of: (**a**) NF-ΚB, (**b**) TNF-α, (**c**) COX-2, (**d**) ICAM-1, (**e**) MMP-9, (**f**) Caspase-3, (**g**) VEGF, and (**h**) PPARγ. Data were expressed as mean value ± SD (*n* = 3). (**a**) significance level at *P* < 0.05 in comparison to the negative control (NC) group, (**b**) significance level at *P* < 0.05 in comparison to the gastric ulcer (GU) group

Figure 4 demonstrated the influence of treatment with PANTO, ADSCs or ADSCs + PANTO on the transcriptional patterns of gastric markers of inflammation; NF-ΚB, TNF-α, COX-2, ICAM-1, MMP-9 and the anti-inflammatory marker PPARγ genes in GU-induced rats. The GU group exhibited a significant up-regulation (*P* < 0.05) of NF-ΚB, TNF-α, COX-2, ICAM-1 and MMP-9 gene expressions along with insignificant down-expression (*P* > 0.05) of PPARγ gene expression as compared to the negative control group. On the opposite side, the GU-afflicted rats treated with PANTO, ADSCs or ADSCs + PANTO exhibited a significant down-expression (*P* < 0.05) of the gastric NF-ΚB, TNF-α, COX-2, ICAM-1 and MMP-9 genes when compared with the GU group. On the contrary, the ADSCs- and ADSCs + PANTO-treated groups showed a significant overexpression (*P* < 0.05) in the gastric PPARγ gene when compared with the GU group.

#### Gene expression of the apoptotic marker caspase-3

Figure 4 revealed that the GU group presented a significant up-regulation (*P* < 0.05) of caspase-3 gene expression as compared to the negative control group. However, the GU-afflicted rats treated with PANTO, ADSCs or ADSCs + PANTO exhibited a significant down-expression (*P* > 0.05) of the gastric caspase-3 gene when compared with the GU group.

#### Gene expression of the pro-angiogenic vascular endothelial growth factor (VEGF)

As shown in Fig. 4, the GU group exhibited insignificant down-expression (*P* > 0.05) of the VEGF gene as compared to the negative control group. On the opposite side, the PANTO-, ADSCs- and ADSCs + PANTO-treated groups displayed a significant overexpression (*P* < 0.05) in the gastric VEGF gene *versus* the GU group.

### Immunohistochemical findings

#### Immunohistochemical staining outcomes of transforming growth factor β1 (TGF-β1)

**Fig. 5:**
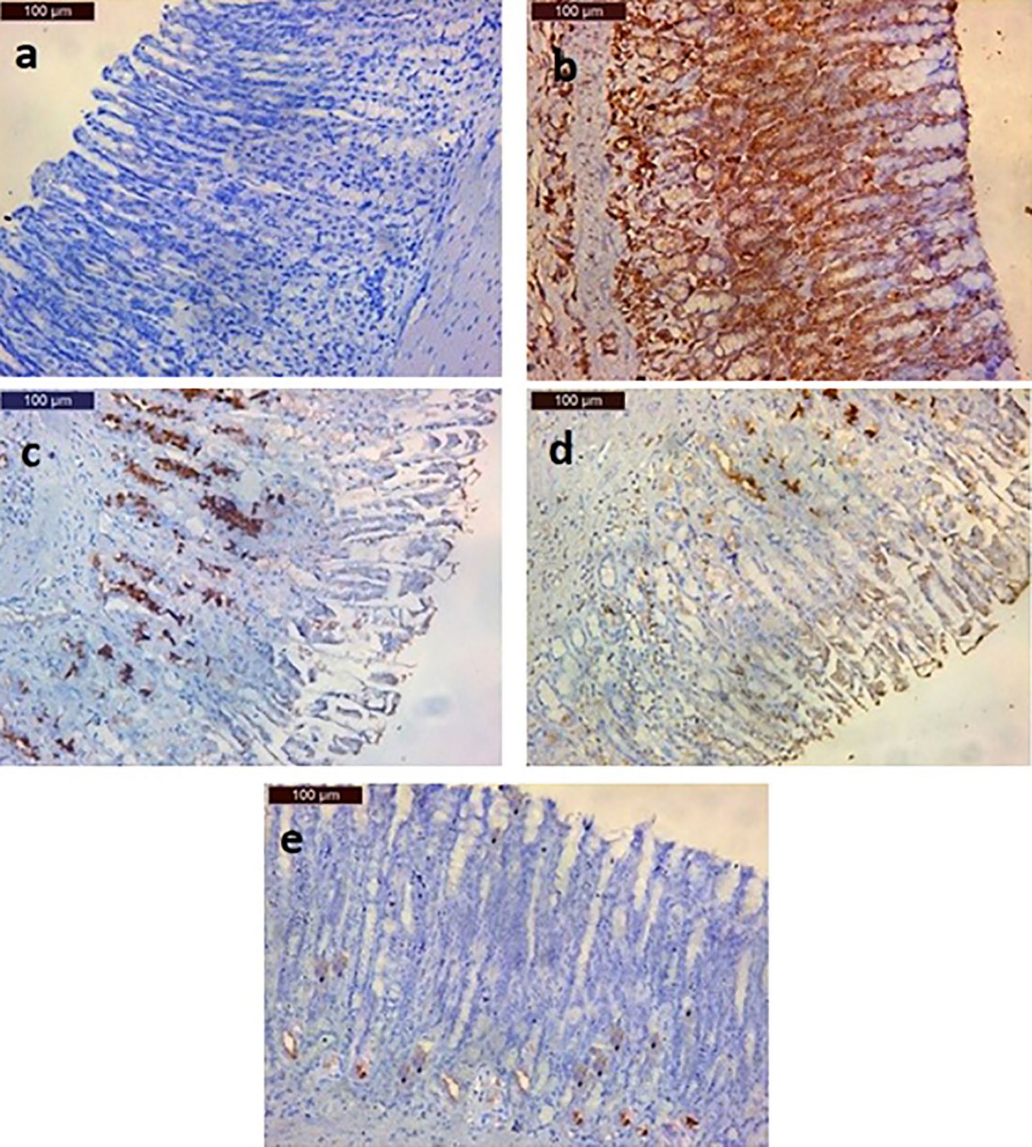
Photomicrographs of gastric tissue sections of rats demonstrating; (**a**) the negative control group with weak reaction for TGF-β1 immunostaining, (**b**) the GU group with intense positive reaction for TGF-β1 immunostaining, (**c**) the PANTO-treated group with moderate immunostaining reaction of TGF-β1, (**d**) the ADSCs-treated group with mild immunostaining reaction of TGF-β1, and (**e**) the ADSCs + PANTO-treated group with weak immunohistochemical reaction of TGF-β1

The immunohistochemical examination of the gastric tissue obtained from the rats in the negative control group revealed weak epithelial TGF-β1 immune reactions (Fig. 5a) while the examination of the TGF-β1 immunohistochemical reaction of the gastric tissue obtained from rats in the GU group showed an intense positive immune reaction (Fig. 5b). Regarding the PANTO-treated group, moderate TGF-β1 immune reaction was observed in the gastric tissue section (Fig. 5c). The Immunohistochemical reaction of TGF-β1 in the gastric tissue taken out from rats in the ADSCs-treated group showed mild TGF-β1 immune reaction (Fig. 5d). However, the gastric tissue derived from rats in the ADSCs + PANTO-treated group showed very weak TGF-β1-immune reaction (Fig. 5e) nearly as the negative control group.

#### Immunohistochemical staining outcomes of epidermal growth factor (EGF)

**Fig. 6:**
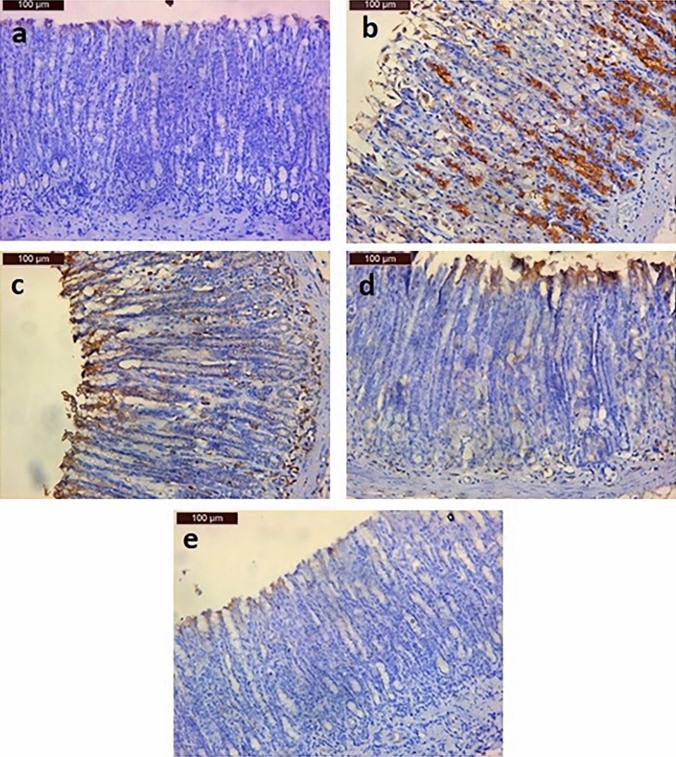
Photomicrographs of gastric tissue sections of rats illustrating; (**a**) the control group with weak immunostaining reaction of EGF, (**b**) the GU group with increased intensity of EGF immunostaining reaction, (**c**) the PANTO-treated group with moderate immunostaining reaction of EGF, (**d**) the ADSCs-treated group with mild immunostaining reaction of EGF, and (immunohistochemical examination of the gastric): the ADSCs + PANTO-treated group with weak immunostaining reaction of EGF

As illustrated in Fig. 6a, the immunohistochemical reaction of EGF in the gastric tissue of negative control rats revealed weak EGF immunoreactivity whereas the immunohistochemical reaction of EGF gastric tissue section obtained from the rat in the GU group showed a noticeable increased intensity of EGF immunohistochemical reactivity (Fig. 6b). For the PANTO-treated group, the intensity of EGF reaction in the gastric tissue showed moderate immunoreactivity (Fig. 6c). However, regarding ADSCs-treated group, EGF immunohistochemical reaction in the gastric tissue showed mild immunoreactivity (Fig. 6d). Finally, the EGF immunohistochemical reaction in the gastric tissue of the ADSCs + PANTO-treated group revealed weak immunohistochemical reaction (Fig. 6e).

#### Immunohistochemical staining outcomes of proliferating cell nuclear antigen (PCNA)

**Fig. 7:**
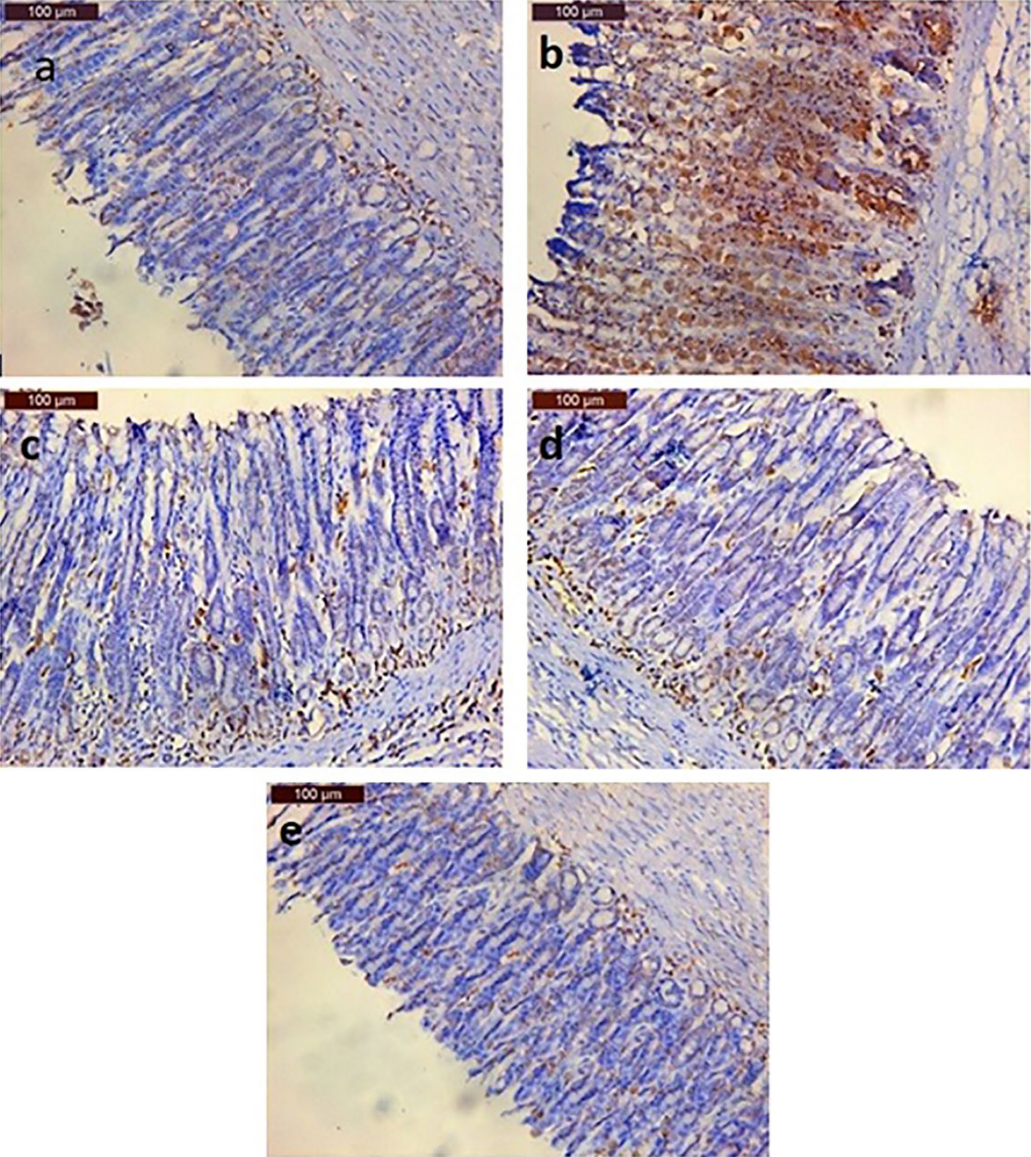
Photomicrographs of gastric tissue sections of rats showing; (**a**) the negative control group with weak PCNA immunohistochemical reaction in the nuclei of the epithelial cells, (**b**) the GU group with an intense immunohistochemical reaction of PCNA in the nuclei of the epithelial cells, (**c**) the PANTO-treated group with moderate immunohistochemical reaction of PCNA in the nuclei of the epithelial cells, (**d**) the ADSCs-treated group with mild immunohistochemical reaction of PCNA in the nuclei of the epithelial cells, and (**e**) the ADSCs + PANTO-treated group with weak immunohistochemical reaction of PCNA in the nuclei of the epithelial cells

As shown in Fig. 7a, the gastric tissue obtained from rats in the negative control group revealed weak PCNA immunohistochemical reaction of the nuclei of the gastric epithelial cells while the PCNA immunohistochemical reaction in the gastric tissue taken out from rats in the GU group showed intense reaction indicated by numerous PCNA positive nuclei (Fig. 7b). Regarding the PANTO-treated group, moderate immunohistochemical reaction was observed in the gastric tissue as indicated by moderate number of PCNA positive nuclei in the epithelial cells (Fig. 7c). Moreover, the ADSCs-treated group showed mild immunohistochemical reaction of PCNA in the gastric tissue as indicated by the mild numbers of PCNA positive nuclei in the epithelial cells (Fig. 7d). Finally, the ADSCs + PANTO-treated group revealed weak immunohistochemical reaction of PCNA in the gastric tissue as indicated by few numbers of PCNA positive nuclei in the epithelial cells (Fig. 7e).

### Histopathological observations

**Fig. 8:**
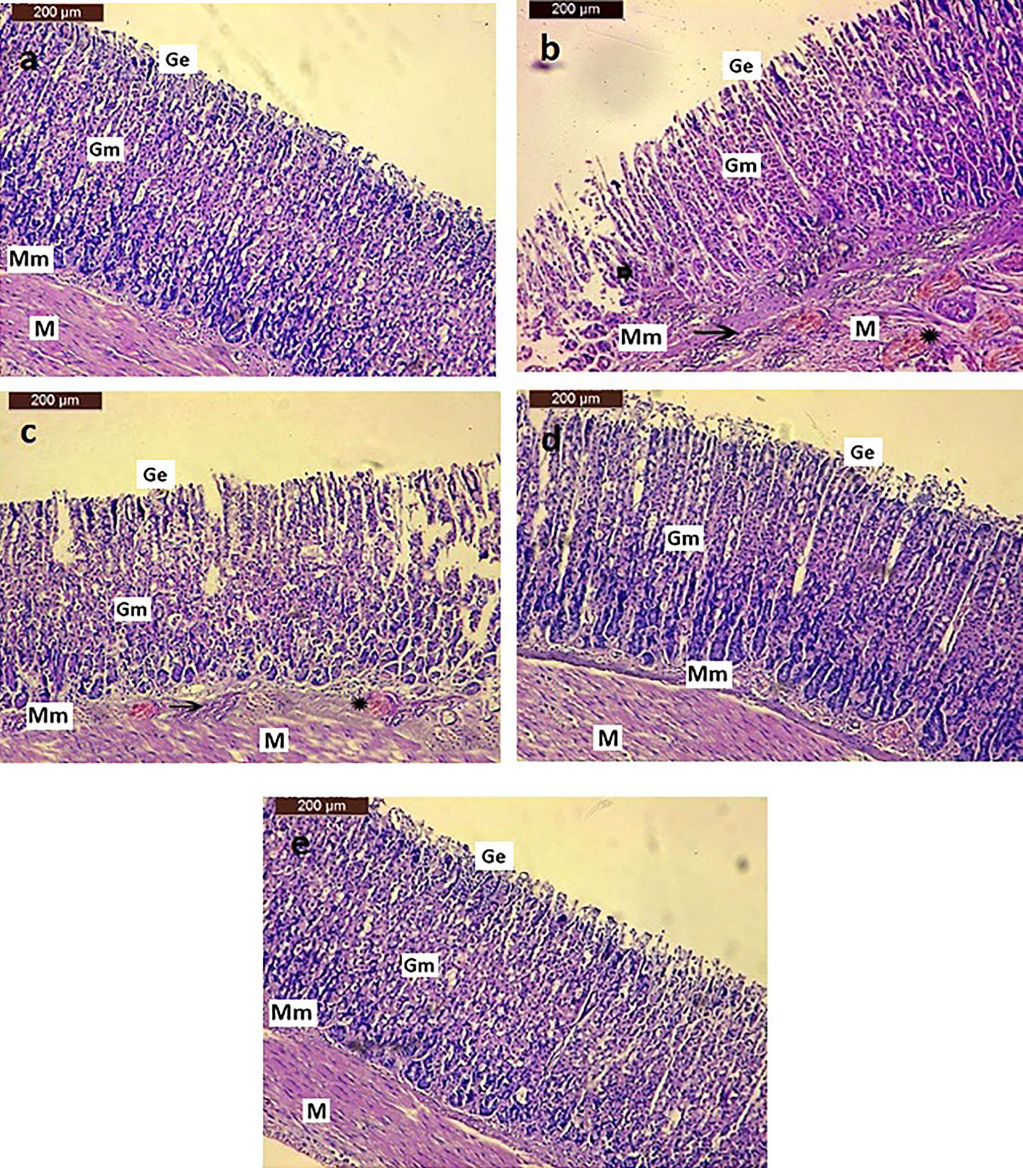
Microscopic investigation of gastric tissue sections of rats showing; (**a**) the negative control group showing normal histological structure of the gastric epithelial cells (Ge), intact gastric mucosal layer (Gm), muscularis mucosa (Mm), submucosa (M) with rounded nuclei, (**b**) the GU group section demonstrates superficial gastric epithelial degeneration and gastric erosions (Ge), necrotic changes, increase in the infiltration of inflammatory cells in gastric mucosa and submucosal regions (arrow), congestion of submucosal blood vessels associated with edema (star), (**c**) the PANTO-treated group section shows moderate improvement of histopathological alterations of the epithelial cells in the mucosal layer (Ge & Gm), mild infiltration of inflammatory cells in gastric mucosal and submucosal regions (arrow), with congestion of submucosal blood vessels (star), (**d**) the ADSCs-treated group section illustrates a good improvement of histopathological alteration of the epithelial cells in the mucosal layer with nearly normal surface mucosal epithelium (Ge); nearly normal gastric architecture (Gm, Mm, & M), (**e**) the ADSCs + PANTO-treated group section shows nearly normal histological structures of the epithelial cells (Ge), intact gastric mucosa layer (Gm), muscularis mucosa (Mm), submucosa (M), with rounded nuclei

Histopathological investigation of gastric tissue of the negative control rats showed typical histological structures of the gastric epithelial cells, intact gastric mucosal layer, muscularis mucosa and submucosa, with rounded nuclei (Fig. 8a). However, the histopathological examination of gastric tissue of the GU rats induced by IND revealed superficial epithelial degenerative and necrotic changes, with mucosal erosions. IND administration also resulted in an increase in the infiltration of inflammatory cells in gastric mucosa and submucosa regions, congestion of submucosal blood vessels associated with edema and pyknotic nuclei. These manifestations were accompanied by an atrophy of gastric mucosa that was observed in some areas (Fig. 8b). Concerning the PANTO-treated group, histopathological investigation of gastric tissue section of rat in this group showed moderate improvement histopathological alteration of the epithelial cells in the mucosal layer with mild infiltration of inflammatory cells in gastric mucosal and submucosal regions in addition to congestion of submucosal blood vessels (Fig. 8c). In addition, the histopathological investigation of gastric tissue of the ADSCs-treated rats displayed a good improvement of histopathological alteration of the epithelial cells in the mucosal layer, reaching nearly normal surface mucosal epithelium; nearly normal gastric architecture (Fig. 8d). Finally, almost as shown in the gastric tissue section of the control rats, the section of the gastric tissue section of rats in the ADSCs + PANTO-treated group showed almost normal histological structures of the epithelial cells, with intact gastric mucosal layer, muscularis mucosa and submucosa, with rounded nuclei (Fig. 8e).

## Discussion

This research aimed to investigate the possible curative effect of ADSCs and PANTO either alone or in combination on IND-induced GU in rats, and to discover the potential mechanisms behind this effect.

Oxidative stress denotes the imbalance between the intracellular redox system and free radicals generation, resulting in the deterioration of lipids, proteins, and DNA structure leading to cell and tissue damage (Fernández-Francos et al. 2021). Oxidative stress has been found the main cause of gastric ulceration by IND (Hafez et al. 2019). AOPPs represent the end-products of a reaction between plasma albumin and chlorinated oxidants, such as hypochlorous acid, and are formed through myeloperoxidase (Ou et al. 2017). Once AOPPs are formed, they can induce tissue damage as a result of the imbalance between the antioxidant and pro-oxidant levels and the accumulation of reactive oxygen species (ROS), which are strong oxidizing agents (Bagyura et al. 2022).

In the current study, the GU group showed a significant increase in serum AOPPs levels, and this result agrees with Ma et al. (2022) who declared that IND significantly up-regulated mucosal and plasma AOPPs levels in the GU group. IND induces the production of large amounts of ROS leading to oxidative stress in the gastric tissue (Bhattacharyya et al. 2014). The high levels of ROS diminish the intracellular content of some antioxidant enzymes and non-enzymatic antioxidants of gastric tissue, resulting in the generation of some protein and lipid peroxidation byproducts, such as MDA and AOPPs (Barboza et al. 2018).

Regarding the PANTO-treated group, our results showed a significant reduction in serum AOPPs level, and this finding correlates with Barboza et al. (2018) who stated that lansoprazole significantly attenuates the level of plasma AOPPs owing to its antioxidant activity. Concerning ADSCs- and ADSCs + PANTO-treated groups, a significant depletion in serum AOPPs level was detected. Many proofs confirm the antioxidant activity of MSCs in a variety of animal disease modeling, which may elucidate their cell protective and anti-inflammatory properties (Stavely and Nurgali 2020). The synergetic effect of the combined treatment of ADSCs and PANTO produced superior recovery from the oxidative stress and its consequences as evidenced by the significant drop in serum level of AOPPs as shown in the present investigation.

The present results demonstrated a significant increase in serum AGEPs level in the GU group which concurs with that of Kotob et al. (2018) who recorded a significant elevation in serum AGEPs level in the GU group. It has been described that GU generation enhances the serum level of high-mobility group box 1 (HMGB1). HMGB1 is a nuclear protein that binds with DNA as a chromatin-associated non-histone protein to support nucleosomes and mediate gene transcription in the nucleus. Upon its secretion into the extracellular environment, it induces inflammatory pathway by promoting various receptors, involving toll-like receptor 4 (TLR4), TLR2, and receptor for advanced glycation end products leading to tissue injury (Nadatani et al. 2013).

Concerning the PANTO-treated group, it exhibited significant depression in serum AGEPs level. This is in harmony with Kotob et al. (2018) who found that treatment with omeprazole before GU initiation in rats causes a significant reduction in serum AGEPs level. It has been found that PPIs prevented thioglycollate-induced peritoneal inflammation. Thioglycollate is activated by the interaction between proteins and the reduced sugars resulting in the production of AGEPs (Li et al. 1997). In the current approach, the ADSCs-treated group displayed significant relief of AGEPs serum level. Han et al. (2017) reported that MSCs could clear AGEPs via inducing autophagy; hence, MSCs may become a perfect autophagy activator in wound healing promotion. ADSCs and PANTO co-treatment heightened the marked decrease in AGEPs serum level in the present study as they act together to prohibit the formation of AGEPs and remove these toxic compounds from the circulation to promote tissue repair.

The current study revealed a significant decline in serum TAC in the GU group and this result comes in line with that of Abu-Raia et al. (2022) who reported that induction of peptic ulcer in rats by IND yields a significant decrease in TAC. This could be ascribed to the respiratory chain suppression leading to the liberation of cytochrome c from mitochondria to the cytosol and subsequent ROS production by IND (Abd El-Rady et al. 2021).

Concerning the PANTO-treated group, it showed significant enhancement in serum TAC, and this finding coincides with that of Tamaddonfard et al. (2019) who stated that the decreased activities of TAC induced by IND are normalized by lansoprazole. Shukla et al. (2014) recorded a potent antioxidant impact of PANTO as these investigators demonstrated a significant increase in the level of the reduced glutathione (GSH) and the activities of SOD and CAT enzymes in animals afflicted with reflux esophagitis suggesting the enhancement of the antioxidant status and the decreased oxidative stress due to treatment with PANTO. Regarding the treatment of GU-bearing rats with ADSCs, this group revealed a significant elevation in serum TAC. It has been reported that MSCs can overcome oxidative stress via the induction of the antioxidant enzymes [SOD1, SOD2, catalase (CAT), and glutathione peroxidase (GPx)], and enhancement of GSH in vitro (Valle-Prieto and Conget 2010) as well as activation of TAC (Al-Massri et al. 2019) in vivo. In the current study, the combination effect of ADSCs + PANTO produced the best effect in augmenting TAC in the serum of the treated rats which could be attributed to the synergy between ADSCs and PANTO to reinforce the total antioxidant defense system.

The findings of the current research showed a significant up-regulation of NF-κB gene in the GU group. The recent study of Ma et al. (2022) demonstrated that IND significantly activated NF-κB. Our result is in harmony with Hafez et al. (2019) who recorded an increase in the gene expression of NF-κB in GU induced by IND in rats. These investigators attributed this effect to the oxidative stress induced by IND as oxidative stress can stimulate transcription factors including NF-κB (Zha et al. 2014).

In the present work, the PANTO-treated group displayed a significant down-regulation of NF-κB gene expression level. In normal conditions, NF-κB dimers are found bound with IκB in the cytoplasm. Upon motivation by NF-κB stimuli, IKK complex is activated and phosphorylates IκB leading to the liberation of NF-κB dimers followed by its transfer to the nucleus where it binds to the promoters of downstream genes including cyclin-D1, iNOS, and COX-2 (Hoesel and Schmid 2013). It has been revealed that NF-κB gene expression level is downregulated following PANTO treatment by inhibiting the activation of IKK complex and preventing p65 nuclear transfer. Moreover, PANTO treatment down-regulates the transcription of NF-κB-related genes, such as cyclin-D1, iNOS, and COX-2, demonstrating the anti-inflammatory response of PANTO (Geeviman et al. 2018). A significant down-regulation of the gene expression level of NF-κB in the ADSCs-treated group has been demonstrated in the present investigation. Emerging evidence has indicated that the ADSCs-treated group experienced significant down-regulation in NF-κB gene expression level; MSCs are involved in the modulation of inflammation. It has been demonstrated that MSCs could suppress the NF-κB signaling pathway by modulating the expression of TLR4 and IκB-α and p65 phosphorylation resulting in the reduction in the levels of inflammation. (Liu et al. 2022). The treatment with a combination of ADSCs + PANTO brought about significant down-regulation of NF-κB gene expression level. This effect could be attributed to the powerful anti-inflammatory activity of the two delegates.

The present investigation showed that the induction of GU by IND significantly up-regulated the expression level of the TNF-α gene. A similar finding has been reported previously in the study of Ahmed et al. (2020) who reported a significant overexpression of the TNF-α gene in rats with GU. IND stimulated the injury of small intestinal in murine, with simultaneous overexpression of TNF-α and MCP-1 (Watanabe et al. 2008).

The down-regulation of TNF-α gene expression level in the PANTO-treated group in the present study is in great agreement with Lee et al. (2021) who demonstrated a down-regulation in TNF-α gene expression level in rats afflicted with GU induced by IND. These investigators explained this finding by the capability of PANTO to up-regulate HO-1 expression which contributes to the anti-inflammatory effect of this drug. The significant down-regulation of the gene expression level of TNF-α in the ADSCs-treated group has been reported in the current work. MSCs accelerate gastric ulcer healing by lowering the inflammatory phase and increasing the proliferative phase of the regeneration process. Furthermore, MSCs inhibited the release of inflammatory mediators and cytokines, thereby reducing the negative impact of inflammation (El-Azab et al. 2018) via the down-regulation of the inflammatory factors including TNF-α (Mohammadzadeh et al. 2014). The group of GU treated with ADSCs + PANTO revealed a significant down-regulation of TNF-α gene expression level. This could be attributed to the effect of each candidate in the expression of TNF-α gene via different pathways.

Our findings revealed up-regulation of COX-2 gene expression level in the GU group, which is consistent with that of Shahin et al. (2018). Davies et al. (1997) observed an up-regulation of COX-2 gene expression following COX-1 inhibition by IND which might be explained as a compensatory reaction to the drop in PGE2 production.

The current finding revealed a significant down-expression of the COX-2 gene in the PANTO-treated group. This result is in accordance with Geeviman et al. (2018) who found that PANTO significantly reduces COX-2, iNOS, and cyclin-D1 gene expression levels. These investigators demonstrated that the down-regulated COX-2 gene expression level by PANTO treatment was attributed to PANTO inhibitory effect on NF-κB pathway via inhibiting IKKα activity and hence hindering IκBα phosphorylation resulting in preventing NF-κB-P50/P65 nuclear translocation which in turn blocks its binding to promoters of target genes including COX-2, leading to its inactivation. The present study recorded a significant down-regulation of COX-2 gene expression level in the ADSCs-treated group. Liu and co-workers (2022) reported that MSCs hindered the NF-κB signaling pathway by inhibiting the phosphorylation of IκB-α and p65 and thus preventing the nuclear translocation of NF-κB dimer and prevent activation of downstream genes including COX-2 genes. Thus, the possible explanation for down-regulation of COX-2 by ADSCs treatment could be due to the suppression of NF-κB activity, which results in suppression of the transcription of target genes including COX-2 resulting in its downregulated expression level. Co-treatment with ADSCs and PANTO produced a significant down-regulation of COX-2 gene expression level due to their anti-inflammatory and antioxidant properties.

In the current approach, the GU group exhibited a significant up-regulation in the ICAM-1 gene expression level. This agrees with the findings of Okada et al. (1998) who noted a significant up-regulation of ICAM-1 gene expression in IND-induced gastritis in rats. These investigators observed over-expression of ICAM-1 and TNF-α genes at the early stages of IND-induced gastritis.

Regarding the PANTO-treated group, it displayed a significant down-regulation in the ICAM-1 gene expression level. A previous study demonstrated that IND markedly increases the expressions of TNF-α, IL-1ß, IL-8, NOX-1, and ICAM-1, whereas PANTO significantly decreases the expression of IND-induced inflammatory mediators (Lee et al. 2012). This might be explained by the ability of PANTO to induce HO-1 expression which could attenuate the level of ICAM-1. The ADSCs-treated group showed down-regulation of ICAM-1 gene expression level. This finding is in accordance with that of the recent study of Liu et al. (2022). Wen et al. (2014) concluded that MSCs reduce the expression of adhesion molecules including ICAM-1 and immunoregulatory molecules via NF-κB signaling pathways. Suppression of NF-κB mRNA transcriptional level has been reported in MSCs transplantation in myocardial infarction (Du et al. 2008). The co-administration of ADSCs and PANTO brought about significant down-regulation of the ICAM-1 gene expression level. This could be explained by the role of ADSCs and PANTO as anti-inflammatory candidates in addition to PANTO as an inducer of HO-1 expression.

The results of the current study revealed a significant up-regulation in MMP-9 gene expression level in the GU group which is in concert with (Sherif et al. 2019). MAPK cascade, namely ERK1/2, closely regulates the transcriptional level of MMP-9 production by phosphorylating the essential serine/threonine residues (Mori et al. 2003). Shahin et al. (2018) observed an increase in the ERK1/2 signaling in IND-treated rats which might be a mediator for the up-regulation of the expression of the MMP-9 gene.

The PANTO-treated group in the present work displayed a significant down-regulation in the MMP-9 gene expression level. This effect of PANTO could be attributed to the down-regulation of TNF-α; the main trigger of MMP-9 gene expression in gastric tissue (Ganguly and Swarnakar 2009). The ADSCs-treated group in the present investigation experienced significant down-regulation in MMP-9 gene expression level. Chen et al. (2014) reported that MSC transplantation reduces the expression of pro-inflammatory cytokines (TNF-α, IL-1β, and IL-6) in a mice model of colitis-associated tumorigenesis as demonstrated by Real-time PCR. The down-regulation of TNF-α gene expression constitutes the mechanism by which ADSCs could down-regulate MMP-9 gene expression level. The combined treatment with ADSCs and PANTO yielded a significant down-regulation of the MMP-9 gene expression level. This result could be attributed to the synergistic impact of the two treatments on TNF- α gene expression.

Caspase-3 is a crucial modulator of apoptosis; in the present investigation, the GU group exhibited a significant up-regulation in the Caspase-3 gene expression level. This result converges with Hafez et al. (2019) who recorded an up-regulation in Caspase-3 gene expression in gastric tissue of the experimental GU model. It has been suggested that TNF-α, which is conspicuously raised in the damaged mucosa (Shahin et al. 2018) and the ROS, which are probably overproduced by the invading inflammatory cells, are crucial for IND-induced gastric mucosal apoptosis (Fouad et al. 2010).

Our results revealed that the PANTO-treated group presented a significant down-regulation in the Caspase-3 gene expression level. This finding corresponds with Tamaddonfard et al. (2019) who stated that lansoprazole produced a significant decrease in Caspase-3 gene expression level induced by IND. Pantoprazole inhibits apoptosis through regulating Bax and Bcl-2 gene expression and cleavage of Caspase-3 protein. Concerning the ADSCs-treated group, it showed a significant down-regulation of the Caspase-3 gene level. Our result concurs with Rashed et al. (2016) who found that administration of BMMSCs to the IND-treated group considerably down-regulated Caspase-3 gene expression level. The co-treatment with ADSCs and PANTO could significantly down-regulate the Caspase-3 gene expression level due to the synergistic effect of both treatments as powerful anti-apoptotic candidates.

The results of the current study revealed an insignificant down-expression of the VEGF gene in the GU group, and this agrees with Abd El-Salam et al. (2019). Antonisamy et al. (2014) showed that the gastric mucosal injury induced by IND administration caused a prominent decrease in VEFG content in the gastric mucosa. Furthermore, it has been detected that GU healing is prolonged due to the decrease of angiogenesis in response to a reduction in expression of VEGF (Harsch et al. 2003).

Regarding the PANTO-treated group, the current findings indicate a significant up-regulation in the expression level of the VEGF gene. Kobayashi et al. (2010) mentioned that the administration of lansoprazole led to the upregulation of VEGF gene expression levels in rats with GU induced by acetic acid. These results suggest the protective role of proton pump inhibitors against GU formation via modulating the expression of growth factors rather than preventing the secretion of gastric acid. Endogenous PG secretion is considered to be one of the mechanisms involved in ulcer healing induced by lansoprazole which may be related to the induction of VEGF expression. Concerning ADSCs treatment, it displayed a significant up-regulation in the VEGF gene expression level. Abd El-Salam et al. (2019) stated that MSCs have significant therapeutic effects in GU via releasing growth factors such as VEGF which induces angiogenesis and keeps the blood supply to the gastric mucosa. The combined treatment with ADSCs + PANTO resulted in significant up-regulation of VEGF gene expression level. This effect of combination therapy could be explained by the capability of ADSCs to express VEGF in addition to the role of PANTO in stimulating PG which is a good inducer of VEGF.

In the current investigation, there is an insignificant down-expression of PPARγ gene in the GU group. El Senosi et al. (2020) reported that induction of GU caused a significant down-regulation in the expression level of PPAR-γ in rats. It has been reported that the elevated levels of TNF-α can down-regulate PPARγ expression (Tanaka et al. 1999). In the present approach, IND could up-regulate TNF-α gene expression level which in turn caused a down-regulation in PPARγ gene expression.

In this study, PANTO treatment brought about significant up-regulation of the PPAR-γ gene expression level. It has been reported that in cultured tracheal human epithelial cells, lansoprazole could decrease the levels of many pro-inflammatory cytokines such as IL-6, IL-8 and TNF-α (Sasaki et al. 2005). This supports the finding of the present investigation as PANTO could down-regulate TNF-α gene expression level. Thus, one can hypothesize that the down-regulation of TNF-α by PANTO leads to the up-regulation of PPARγ gene expression. The ADSCs-treated group in the current approach displayed significant up-regulation in PPARγ gene expression level. MSCs have been found to suppress T effector cells and many other immune cells, while stimulating regulatory T cells, hence decreasing the secretion of pro-inflammatory cytokines including TNF-α (Yan et al. 2018). Thus, the anti-inflammatory property of ADSCs enables them to down-regulate TNF-α gene expression level in gastric tissue as shown in the present findings. This effect of ADSCs may be the underlying mechanism by which ADSCs can up-regulate PPARγ gene expression level in gastric tissue in the current investigation. The co-administration of ADSCs + PANTO produced significant up-regulation of the PPAR-γ gene expression level. This effect could be attributed to the synergistic impact of the two candidates in inhibiting the production of TNF-α via down-regulation of TNF-α gene expression.

In the present approach, the gastric tissue of rats in the negative control group revealed a weak epithelial TGF-β1 immune reaction while examination of the TGF-β1 immunohistochemically stained section of rat gastric tissue from the GU group showed an intense positive immune reaction of TGF-β1. This result could be explained by that at the early phase of ulcer healing, many macrophages and some polymorphonuclear leukocytes in the region of the ulcer bed produced TGF-β1 which stimulates chemoattraction of leukocytes (Border and Ruoslahti 1992), migration of macrophages (Postlethwaite et al. 1987) and fibroblasts (Wahl et al. 1987). Therefore, TGF-β1, produced by these cells, may be involved in the migration of inflammatory cells and fibroblasts in an autocrine and/or paracrine manner, thereby leading to the construction of granulation tissues.

In the present study, the immunohistochemical results revealed moderate immunoreactivity toward TGF-β1 in the gastric tissue of the PANTO-treated group. Similarly, a very recent study (Allam et al. 2023) reported that omeprazole causes TGF-β/Smad signaling activation in the rat kidney. Also, the immunohistochemical investigation of TGF-β1 in gastric tissue of the ADSCs-treated group showed mild TGF-β1 immune reaction. Liu et al. (2015) reported that TGF-β1 expression is significantly increased in the ADSCs-treated group, indicating the important role of TGF-β1 during the healing of gastric injury. In the regenerative and remodeling phases during wound healing, TGF-β1 increases production of extracellular matrix and collagen I, promotes regeneration of blood vessels, and enhances wound tensile strength and healing (Douglas 2010). The gastric tissue of rat in the ADSCs + PANTO-treated group showed a weak TGF-β1 immune reaction, nearly as the negative control group, indicating almost a complete healing of GU.

In the current study, the negative control group revealed weak EGF immunohistochemical reactivity of the gastric epithelium while the examination of immunohistochemically EGF-stained section of gastric tissue of rat in the GU group displayed increased intensity of EGF reaction. Our result showed parallelism with that of Aupperlee et al. (2014) who mentioned that EGF expression in the GU group is greater than that of the control group. They claimed that EGF's interaction with its cell surface receptor (EGFR) has a big role in ulcer healing. Moreover, Our results coincide with that of Choi et al. (2008) who reported that after the induction of GU, the expression of EGFR mRNA and protein reached a maximum level at 12 and 24 h, respectively, as it plays a key role in the early stages of ulcer healing. Seven days later, they reached the normal levels of the control group. In the present work, in the PANTO-treated group, 14 days after the induction of GU, the intensity of EGF expression in the gastric tissue showed moderate immunopositivity, indicating the reasonable effect of PANTO toward ulcer healing. Hritz et al. (2005) mentioned that after the administration of PPI, a strong positive EGFR immunoreactivity was observed predominantly in some mucous neck cells of the proliferative zone compared to the weaker staining density in parietal cells, and that the EGFR immunoreactivity was localized not only on the basolateral membrane of these cells, but also appeared in the cytoplasm and the supranuclear area.

Concerning the ADSCs-treated group, it showed a mild immune reaction toward EGF in the gastric tissue. This finding corresponds with Abd-Elmenm et al. (2016) who reported that MSCs are greatly effective in treating GU as they secrete growth factors, including EGF and VEGF, stimulating angiogenesis and preserving vascular permeability. The weak immunoreactive EGF in gastric tissue of rat in the group treated with a combination of ADSCs and PANTO indicates the promotion of healing process reaching the normal feature of the negative control group with weak immunoreactivity for EGF.

Proliferating cell nuclear antigen (PCNA) is a nuclear protein expressed during cell proliferation (Vasconcelos et al. 2010). In the current study, the control group revealed weak PCNA immunohistochemical reaction of the nuclei of the epithelial cells of gastric tissue of rats while the expression of immunohistochemically PCNA-stained section of the gastric tissue of rat from the GU group showed many PCNA positive nuclei of the epithelial cells of the gastric tissue. This result is consistent with recent studies showing that the gastric mucosa has strong immunoreactivity to PCNA dispersed throughout all gastric mucosal glandular (Alazzouni et al. 2020) and epithelial cells (El-Kerdasy and Mousa 2021). The intense immunoreactivity to PCNA in the gastric tissue of rat in the GU group can be described as PCNA is a major indicator of tissue proliferation that occurs immediately after GU induction.

In our study, the PANTO-treated group showed moderately immunoreactive PCNA in the epithelial cells of the gastric tissue of rats. Similarly, Alazzouni et al. (2020) found that PANTO-treated rats’ glandular gastric mucosa cells have moderate immunoreactivity to PCNA, indicating their attempts to restore a normal level of proliferation. Concerning the immunohistochemical staining of PCNA in the gastric tissue of rats in the ADSCs-treated group, it revealed mild positive nuclei in the epithelial cells. This finding comes in line with El-Kerdasy and Mousa (2021) who reported that the ADSCs-treated group showed mild PCNA expression in the neck cells as compared with the aspirin-induced GU group as indicated in the immunohistochemical examination. MSCs enable to rebuild injured tissue and normalize the proliferative process following re-epithelialization, differentiation, and success in GU healing (Alazzouni et al. 2020). In the current study, the ADSCs + PANTO-treated group showed weak immunoreactive PCNA positive nuclei in the epithelial cells of gastric tissue reaching up to normal levels in gastric tissues suggesting that the combination of both treatments promoted healing of the GU, which is apparent in the histopathological sections in the current work.

The histopathological investigation in our study showed that the GU group revealed severe lesions with superficial epithelial degenerative and necrotic changes as well as mucosal erosions. Also, GU induced by IND displayed an increase in the infiltration of inflammatory cells in gastric mucosal and submucosal regions with congestion of submucosal blood vessels associated with edema and pyknotic nuclei. Moreover, atrophy of gastric mucosa was observed in some areas. Similar histopathological findings were reported by another study performed by Sherif et al. (2019). These histopathological features are likely caused by the polar lipids in IND, which have a strong affinity for the lipophilic regions of cell membranes leading to their disruption. This lessens the mucosal coat hydrophobicity, which makes it easier to damage. IND also changes the fluidity of the membrane, which is a crucial factor in the development of gastric mucosal lesions (Suleyman et al. 2010).

In our current investigation, treatment with PANTO resulted in moderate improvement of histopathological alteration of the epithelial cells in the gastric mucosa, with mild infiltration of inflammatory cells in gastric mucosal and submucosal regions, accompanied by congestion of submucosal blood vessels. These manifestations are consistent with those of Ma et al. (2022) who found that omeprazole could enhance GU healing as observed in the histological sections. Moreover, Alazzouni et al. (2020) found that PANTO-treated rats displayed partial damage surface mucous cells, pyknotic, karyolitic parietal cells and karyorrhexis nuclei among chief cells in the basal region. The histopathological examination of the gastric tissue section of rat in the ADSCs-treated group showed amelioration of the gastric histopathological changes induced by IND resulting in a good improvement of histopathological alteration, and nearly normal surface mucosal epithelium, besides the normal gastric structure. These observations are in line with those reported by Xia et al. (2019) who observed that the transplantation of ADSCs promoted re-epithelization, angiogenesis, and healing of NSAID-induced GU. Concerning the ADSCs + PANTO-treated group, the histopathological examination revealed a superior ulcer healing effect as compared with their separate treatment where sections of the gastric tissue showed nearly normal histological structures of the epithelial cells, with intact gastric mucosa layer, muscularis mucosa, submucosa, with rounded nuclei confirming their synergistic effect in enhancing the gastric tissue healing.

## Limitations of the study

Although our study reported that the combined treatments of PANTO and ADSCs exert their healing effect against gastric ulcer via inhibiting the NF-κB pathway and its target genes, more verifications are required to confirm the engagement of this inflammatory pathway via measuring the expression levels of inflammatory and apoptotic proteins. Therefore, further studies are needed to provide more preclinical evidence about the exact mechanisms of such combined treatments against gastric ulcer before they can be utilized in clinical application.

## Conclusion

From the current line of evidence, it was concluded that the co-treatment with ADSCs and PANTO could impact momentous protection against the gastric ulcer by restoring the balance between oxidants and antioxidants (as shown by the significant drop in serum AOPPs and AGEPs levels with the significant increase in serum TAC as compared with the GU group), improving vascularization of gastric tissue (as revealed by the significant overexpression in the gastric VEGF gene *versus* the GU group), recovering the inflammation (as shown by the significant down-expression of the gastric tissue NF-ΚB, TNF-α, COX-2, ICAM-1 and MMP-9 genes with the significant overexpression in the gastric PPARγ gene when compared with the GU group), apoptosis (as indicated by the significant down-expression of the gastric caspase-3 gene when compared with the GU group), and tissue remodeling, repairing and cell proliferative effects (as shown by the weak immune reactions for TGF-β1, EGF and PCNA), and in turn, accelerating the healing process of gastric tissue.

## Implications and future directions

The promising curative action of a combination of ADSCs and PANTO against IND-induced gastric ulcer may have a significant advantage in: (1) improving the therapeutic outcomes of the pharmacologically feasible medication, and (2) generating a fruitful base for the setup of a cell therapy beside the conventional treatment for accelerating the quality of ulcer healing in patients with gastric ulcer.

## Data Availability

The datasets generated and analyzed during the current study are available from the corresponding author on reasonable request.

## Abbreviations

ADSCs: Adipose tissue-derived mesenchymal stem cells
AGEPs: Advanced glycation end products
AOPPs: Advanced oxidation protein products
COX-2: Cyclooxygenase-2
EGF: Epidermal growth factor
GU: Gastric ulcer
ICAM-1: Intercellular adhesion molecule-1
IND: Indomethacin
MMP*-*9: Matrix metallopeptidase 9
MSCs: Mesenchymal stem cells
NF-κB: Nuclear factor kappa-light-chain-enhancer of activated B cells
NSAIDS: Non-steroidal anti-inflammatory drugs
PANTO: Pantoprazole
PCNA: Proliferating cell nuclear antigen
PGE2: Prostaglandin E2
PPARγ: Peroxisome proliferator-activated receptor gamma
PPIs: Proton pump inhibitors
TAC: Total antioxidant capacity
TGF-β1: Transforming growth factor β1
TNF-α: Tumor necrosis factor alpha
VEGF: Vascular endothelial growth factor
